# Microbial community diversity and function analysis of *Aconitum carmichaelii* Debeaux in rhizosphere soil of farmlands in Southwest China

**DOI:** 10.3389/fmicb.2022.1055638

**Published:** 2022-12-15

**Authors:** Tingting Pu, Jie Liu, Jingjing Dong, Jun Qian, Zhongyu Zhou, Conglong Xia, Guangfei Wei, Baozhong Duan

**Affiliations:** ^1^College of Pharmaceutical Science, Dali University, Dali, China; ^2^Key Laboratory of Beijing for Identification and Safety Evaluation of Chinese Medicine, Institute of Chinese Materia Medica, China Academy of Chinese Medical Sciences, Beijing, China

**Keywords:** *Aconitum carmichaelii* Debeaux, microbial community, biogeography, co-occurrence network, soil physicochemical properties

## Abstract

Understanding how microbial communities affect plant growth is crucial for sustainable productivity and ecological health. However, in contrast with the crop system, there is limited information on the microbial community associated with the medicinal plant. We observed that altitude was the most influential factor on the soil microbial community structures of *Aconitum carmichaelii* Debeaux. For community composition, bacterial reads were assigned to 48 phyla, with Proteobacteria, Acidobacteriota, and Actinobacteriota being the dominant phyla. The fungal reads were assigned to seven phyla, and Ascomycota was the predominant phylum detected in most groups. The four dominant phyla were categorized as keystone taxa in the co-occurrence networks, suggesting that they may be involved in soil disease suppression and nutrient mobility. Bacterial co-occurrence networks had fewer edges, lower average degree, and lower density at YL1, HQ1, HQ2, BC, and DL than fungal networks, creating less intricate rhizosphere network patterns. Furthermore, the bacterial and fungal communities showed strong distance decay of similarity across the sampling range. Overall, this study improves our understanding of regulating rhizosphere microbial communities in soil systems and also provides potential production strategies for planting *A. carmichaelii*.

## Introduction

Aconiti Lateralis Radix Praeparata (ALRP) is a traditional Chinese medicine that has been applied for more than 1,000 years in China (Judith et al., 2009; Mao et al., 2021). It originates from the dried roots of *Aconitum carmichaelii* Debeaux, according to the Chinese Pharmacopoeia 2020 edition (Chinese Pharmacopoeia Commission, 2020). This medicine is most notably an ingredient of the traditional Chinese prescriptions “Fu Zi Li Zhong Wan,” “Si Ni Tang,” “Da Huang Fu Zi Tang,” and “Gui Fu Di Huang Wan” (Chen et al., 2011). It plays an important clinical role in treating cardiogenic shock associated with acute myocardial infarction, rheumatism, bruises and injuries, acute or chronic bacterial dysentery, and enteritis (Zhou et al., 2015; Zhang et al., 2020). Due to their remarkable curative effects, there is an increasing demand for this medicine (Fu et al., 2021). Currently, *A. carmichaeli* is cultivated in the southwest region of China as a commercial crop, and most of them are grown in Yunnan province (Xia et al., 2021). However, there is a growing concern that emphasis on below-ground traits during cultivation inadvertently disrupted root morphology, carbon (C) sequestration, and microbial importance for sustainable production (Eaton et al., 2021). Similarly, soil sickness has become a problem in the production of many annual crops, which causes a majority of soil-borne crop diseases, such as root wilt, damping off, and root rot (Huang et al., 2013; Li et al., 2014). This factor is a significant constraint to achieving the required increase in agricultural production and maintaining the high quality of *A. carmichaeli* (Ying et al., 2012; Huang et al., 2013), which seriously threatens the safety of the clinical application and ecological sustainability.

It is well known that rhizosphere microorganisms play a crucial role in disease suppression and the production of plant-growth-promoting hormones and compounds, which directly impact plant health (Mendes et al., 2013; Schlatter et al., 2022), and they are also the key drivers in controlling organic matter mineralization, soil formation, and nutrient availability (Rillig and Mummey, 2006; Xu et al., 2014). Thus, understanding microbial communities’ composition, structure, dynamics, and ecological function is critical in controlling plant health and productivity. To date, many researchers have proved that microbial biogeography could reveal the relationship between the microorganisms and their habitat and could assist scientists or land managers in utilizing microbial communities to increase plant productivity, reduce plant disease, and enhance soil health (Kourtev et al., 2002; Wu et al., 2011). Meanwhile, co-occurrence network analyses have been used to explore the interaction patterns among microbial species in many different environments and identify possible keystone populations in the community (Lynch and Neufeld, 2015), which help to predict functional roles, habitat affinities, and shared physiologies (Ramirez et al., 2018). Although previous studies have discussed the differences in diversity and composition of microbial communities from different growing regions (Qiu et al., 2021), no studies have analyzed the interactions among microbial communities, soil physicochemical properties, and bioactive ingredients. And the coupling relationship between the microbial community structure and their function remains unclear.

This study aimed to evaluate the effect on soil physicochemical properties and microbial community structure of *A. carmichaelii* by characterizing: (1) plant growth and radix quality, (2) soil potential of hydrogen (pH), organic matter (OM), ammonium nitrogen (NH^4+^), phosphorus (AP), and available potassium (AK) contents, (3) diversity and structure of microbial populations in the rhizosphere soils; and (4) the relationship of plant–soil-microbe-ingredient. Herein, we collected rhizosphere soil and root samples from six *A. carmichaeli* growing regions in southwest China, and internal transcribed spacer (ITS)/16S rRNA were employed to characterize the soil microbial diversity and community composition. Redundancy analysis (RDA) was also conducted to evaluate the relationships between soil properties and the relative abundance of microbial taxa. Meanwhile, the linear discriminant analysis of effect size (LEfSe) was used to compare the changes in microbial communities and identify the biomarkers of each sample site at taxonomic levels. And the geographical and environmental distance-decay relationships (DDRs) and co-occurrence networks were constructed for bacteria and fungi, respectively. Besides, the bioactive ingredients of the ALRP were determined by high-performance liquid chromatography (HPLC). And Spearman’s correlation was used to examine the correlations between soil physicochemical properties and bioactive ingredients. The present study offers potentially valuable insight into the beneficial effects on soil physicochemical properties, microbial communities, the growth of *A. carmichaelii*, and radix quality in different regions.

## Materials and methods

### Site area and sampling collection

**Figure 1:**
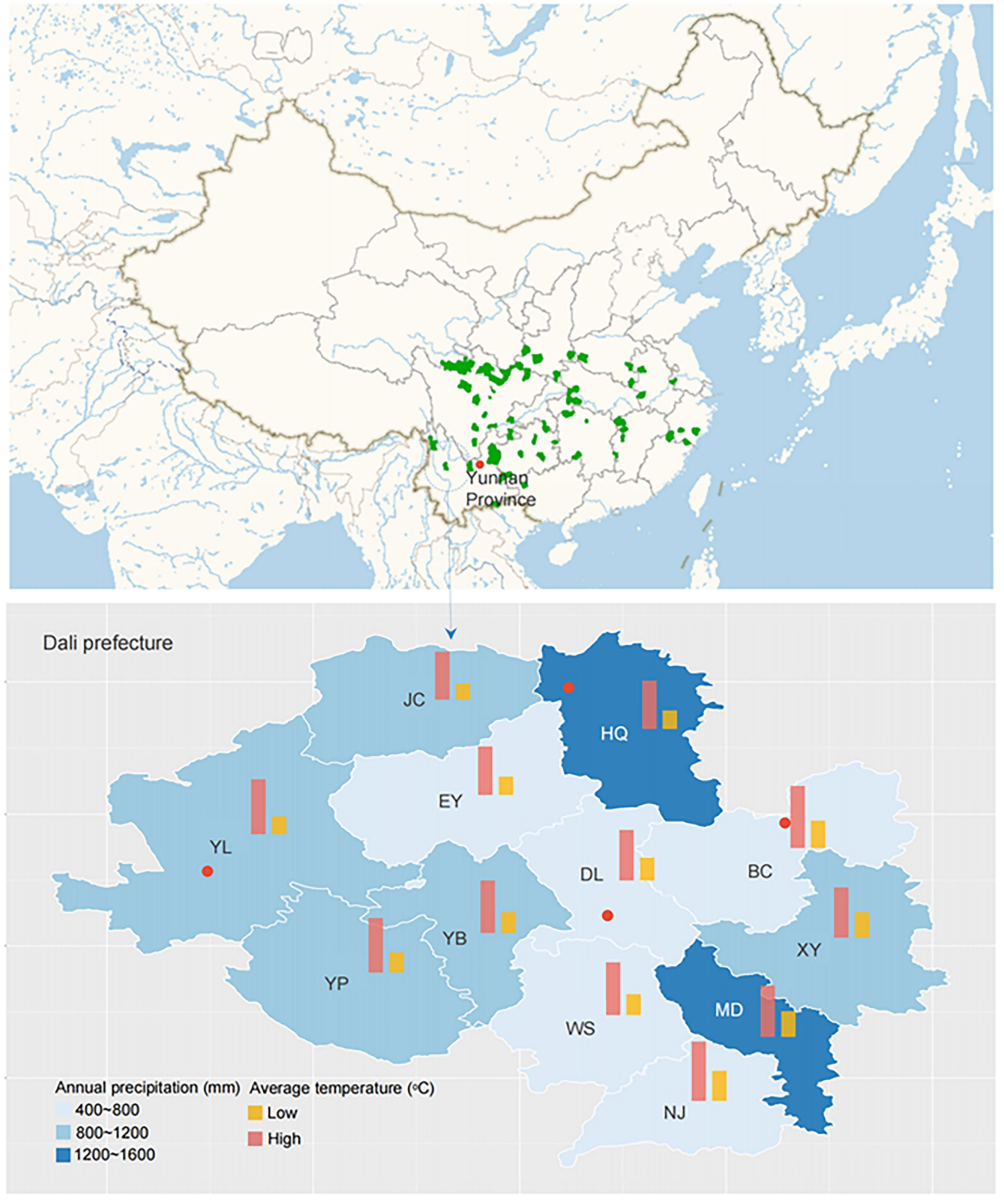
Localization of the sampling sites (the red points represent the collection place: YL, BC, HQ, and DL).

The study area was located in Dali prefecture, Yunnan Province, China (Figure 1). The climate is humid continental, with mean annual temperatures ranging from 9.5 to 22.2°C and a mean annual precipitation of 791 mm. Six sampling sites, including Yunlong county (YL1 and YL2), Bingchuan county (BC), Heqing county (HQ1 and HQ2), and Dali city (DL), were randomly selected from the study area at different latitudes (1,965–2,985 m, Table 1).

**Table 1:** The detailed description of soil sample collection and characteristics.

Sites	Yonglong (YL1)	Yonglong (YL2)	Bingchuan (BC)	Heqing (HQ1)	Heqing (HQ2)	Dali (DL)
Soil type	Black soil	Yellow-brown-earth	Black soil	Black soil	Yellow-brown-earth	Black soil
Longitude	E99.21	E99.21	E101.14	E100.12	E100.12	E100.36
Latitude	N26.12	N26.12	N26.63	N26.80	N26.80	N26.01
High average temperature (°C)	24	24	27	21	21	22
Low average temperature (°C)	8	8	12	8	8	10
Annual precipitation (mm)	827	827	724	1,204	1,204	757
Altitude (m)	2060	2060	2,538	2,985	2,985	1965

The rhizosphere soil and root samples were collected in September 2020. For each site, rhizosphere soils from the five sampling points were consolidated into a single composite sample, making three replicates (25 cm in diameter, 5–15 cm in depth). Rhizosphere soil was placed into bags by uprooting the plants and shaking loose soil from the root system. Any remaining soil adhering to the roots was considered rhizosphere soil. The fresh and moist rhizosphere soil was passed through a 0.84 mm mesh sieve, homogenized using a hand trowel, and then immediately transported to the laboratory in covered plastic boxes at 4°C. The homogenized soil was divided into two portions, one portion was applied for soil physiochemical properties analysis, and the other was used for DNA extraction at −80°C. The roots of *A. carmichaelii* were washed with tap water and used to determine the content of chemical components.

### Soil physicochemical properties analysis

Rhizosphere soil was air-dried at 25°C for 5 days, grounded, and passed through a 0.84 mm sieve. The air-dried soil was measured to determine the pH, OM, AP, AK, NH^4+^, Boron (B), Copper (Cu), Iron (Fe), Manganese (Mn), and Zinc (Zn). Soil pH was measured using a pH meter electrode (Affymetrix Inc., Santa Clara, CA, United States). AP, AK, NH^4+^, and OM were determined using a soil chemical fertilizer fast detector (Shandong Lainde Intelligent Technology Inc., Shandong, China). Five elements, including Zn, Fe, Mn, Cu, and B, were determined by inductively coupled plasma optical emissions spectroscopy (ICP-OES 3800, Perkin Elmer Inc., Waltham, United States).

### Ingredients analysis

#### Chemicals and reagents

Benzoylmesaconine (No. 19032406), benzoylaconine (No. 18110710), benzoylhypaconine (No. 19032807), mesaconitine (No. 19080210), aconitine (No. 18110905), and hypaconitine (No. 18111308; purity > 98%) were purchased from Must Biotechnology Co., Ltd. (Chengdu, China). Acetonitrile and ammonium acetate were obtained from Merck (Darmstadt, Germany, 20171013) and Comio Chemical Reagent Co., Ltd. (Tianjin, China, C11805665), respectively. Other chemicals and solvents used were analytical grades.

#### Instrumentation and chromatographic conditions

HPLC analysis was performed on an Agilent 1,260 Infinity system (Agilent Technologies, Palo Alto, CA, United States) equipped with a quaternary pump, an autosampler, a degasser, and a diode array detector (DAD, G7115A). The HPLC column was a ZORBAX SB-C_18_ (5 μm, 4.6 × 250 mm, Agilent, Milford, MA, United States). HPLC analysis was carried out using a mobile gradient phase of acetonitrile (A) and 0.1 mol·L^−1^ ammonium acetate (B) at 30°C. A linear gradient was run as follows: 0–30 min (10–26%, A), 30–50 min (26–35%, A), 50–65 min (35–48%, A), 65–70 min (48–50%, A). The flow rate was 1 ml·min^−1^, the DAD detector was set at 235 nm, and the sample injection volume was 10 μl.

#### Preparation of the sample solution

All root samples were dried at room temperature for 5 days before use, and each dried sample was ground to a fine powder (0.35 mm) using a pulverizer. An accurately weighed sample of 2.0 g powder and extracted by sonication with 50 ml of isopropanol-ethylacetate (1:1) for 60 min, followed by centrifugation for 15 min at 3000 rpm. The supernatant was collected in a volumetric flask and evaporated using a water bath with a temperature of 50°C. The dried extract was dissolved in 2 ml of isopropanol and trichloromethane (1:1), followed by filtration. Subsequently, the filtrate was passed through a 0.22 μm membrane and injected into the HPLC system for analysis. The results of precision and stability tests can be found in our previous study (Liu, 2021).

### DNA extraction and Illumina sequencing

The total DNA was extracted from 500 mg of rhizosphere soil samples using an E.Z.N.A.® Soil DNA Plant Kit (Omega Bio-Tek, Norcross, GA, United States). 16S and ITS sequences were amplified with the 515F/907R and ITS1F/2R primer pairs, respectively (Adams et al., 2013). The amplification system contained 4 μl of 5 × FastPfu Buffer, 2 μl of 2.5 mM dNTPs, 0.8 μl of each primer (5 μM), 0.4 μl of FastPfu polymerase, 10 ng of template DNA, and was then supplemented with ddH_2_O to reach 20 μl. The PCR cycling conditions included an initial cycle of 95°C for 5 min, 25 cycles of 95°C for 30 s, annealing at 55°C for 30 s, elongation at 72°C for 45 s, followed by a final extension at 72°C for 10 min. Amplicons were extracted from 2% agarose gels and purified using the AxyPrep DNA Gel Extraction Kit (Axygen Biosciences, Union City, CA, United States). Finally, amplicons were sequenced by the Illumina MiSeq platform (Shanghai Biozeron Technology Co., Ltd.).

### Sequence data processing

The raw reads were demultiplexed, quality filtered, and merged using Quantitative Insights Into Microbial Ecology (QIIME, version 1.9.0) and the fast length adjustment of the short reads (FLASH, version 1.2.11). The clean data were clustered into operational taxonomic units (OTUs) at 97% sequence similarity (Edgar, 2013). The chimerical sequences were identified and deleted using the UCHIME technique. OTUs were aligned to the Silva 16S rRNA and Unite ITS gene reference databases. Taxonomy was assigned using the Ribosomal Database Project classifier (Wang et al., 2007) with GreenGenes 13.8 and UNITE database reference databases for bacteria and fungi, respectively. The relative abundance was observed at the phylum, class, order, family, genus, and species levels.

### Statistical analysis

All analyses were performed using the package “vegan” in R version 3.6.1 (R Foundation for Statistical Computing, Vienna, Austria) and SPSS 25.0 (SPSS for Windows, United States). Alpha diversities, including Chao, Simpson, and Shannon, were calculated using Mothur software version 8 (Edgar et al., 2011) and tested using Kruskal-Wallis analysis (Liang et al., 2020). Microbial community composition was analyzed using analysis of similarity (ANOSIM) and visualized using principal coordinates analysis (PCoA; Emmert et al., 2021). For bacterial and fungal communities, Bray-Curtis, weighted UniFrac (WU), and unweighted UniFracto (UU) distance matrices were analyzed. The relative abundance of taxa was plotted into a graph using a heatmap. Additionally, LEfSe was used to identify biomarkers (Segata et al., 2011). RDA was conducted to determine the relationship between soil physicochemical variables and the community composition of the soil (Jennifer et al., 2019). Spearman rank correlations were used to test for significant relationships between each pair of OTUs (Barberan et al., 2012). Co-occurrence networks were constructed separately for bacterial and fungal taxa using the 100 most abundant taxa using Spearman correlation matrices (Kelly et al., 2022). The DDR of community similarity across environmental and geographical distances was compared using analysis of covariance (Nekola and White, 2004), and the figures were plotted using ggplot2 (Wickham, 2009). Mantel testing was used to determine significant correlations between Bray-Curtis community dissimilarities, environmental factors, and geographic distances.

## Results

### Microbial diversity and structure

**Figure 2:**
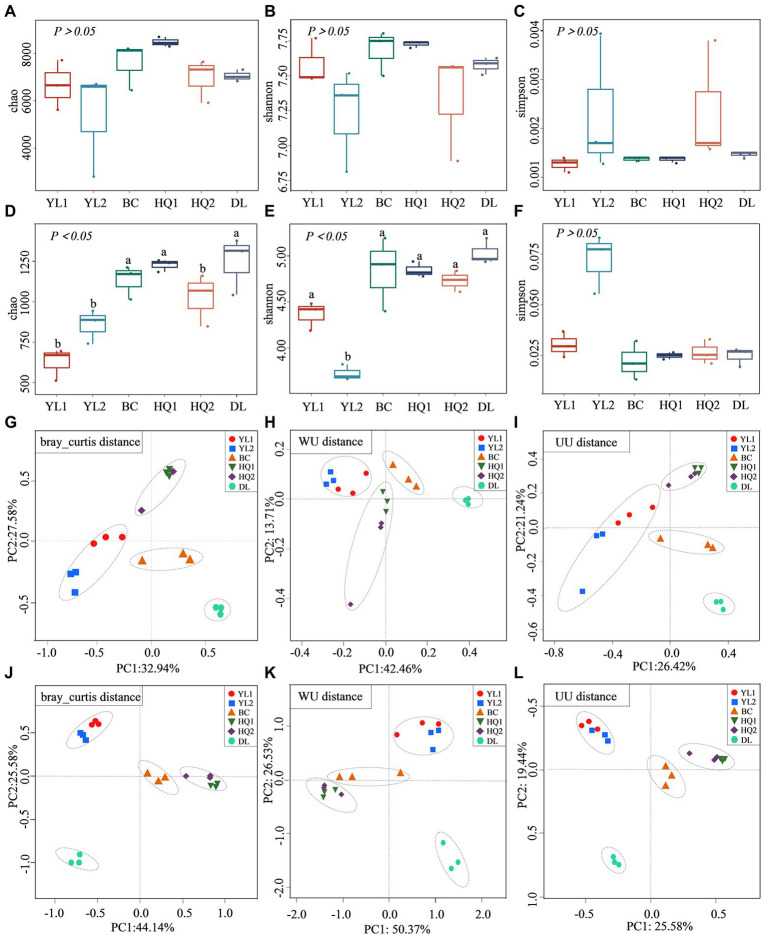
Alpha and beta diversity of microbial communities. **(A–F)** The alpha diversity index of Chao **(A)**, Shannon **(B)**, and Simpson **(C)** for the bacterial community; the alpha diversity index of Chao **(D)**, Shannon **(E)**, and Simpson **(F)** for the fungal community, different letters on the bars indicate significant differences (*p* < 0.05). **(G–L)** the beta diversity of the bacterial community based on the Bray-Curtis **(G)**, WU **(I)**, and UU distances **(H)**; the beta diversity of the fungal community based on the Bray-Curtis **(J)**, WU **(K)**, and UU distances **(L)**.

The alpha diversity (Chao, Shannon, and Simpson) was calculated across all soil samples (Figure 2). For bacterial communities, the results of this assay showed that there were no significant differences among sites for the three indices (*p* > 0.05). As illustrated in Figures 2A-C, the Chao and Shannon were higher in HQ1 compared to other sites, while the Simpson index was higher in YL2 than in other sites. For fungal communities, there were significant differences among sites for Chao and Shannon. The result showed that the Chao was significantly higher in DL, BC, and HQ1 than that in YL1, YL2, and HQ2 (*p* < 0.05), and the Shannon ranked in the order of DL > BC > HQ1 > HQ2 > YL1 > YL2 (Figures 2D–F). Notably, although there is no significant difference in Simpson, the values of Simpson were higher in YL2 (0.072) than BC (0.022). Besides, the variation of beta diversity among sites was visualized using Bray-Curtis, WU, and UU distances. The results indicated that the microbial community structures and compositions significantly differed between sample sites. As shown in Figures 2G–L, the YL1, YL2, BC, HQ1, and HQ2 were clustered together, whereas the DL were positioned far from other locations. In parallel, ANOSIM analysis suggested that the bacterial and fungal communities were significantly different in the six sample sites (bacterial: R^2^ = 0.72, *p* = 0.01, R^2^ = 0.75, *p* = 0.01, R^2^ = 0.82, *p* = 0.001; fungi: R^2^ = 0.92, *p* = 0.01, R^2^ = 0.844, *p* = 0.01, R^2^ = 0.95, *p* = 0.001).

### Microbial community composition

**Figure 3:**
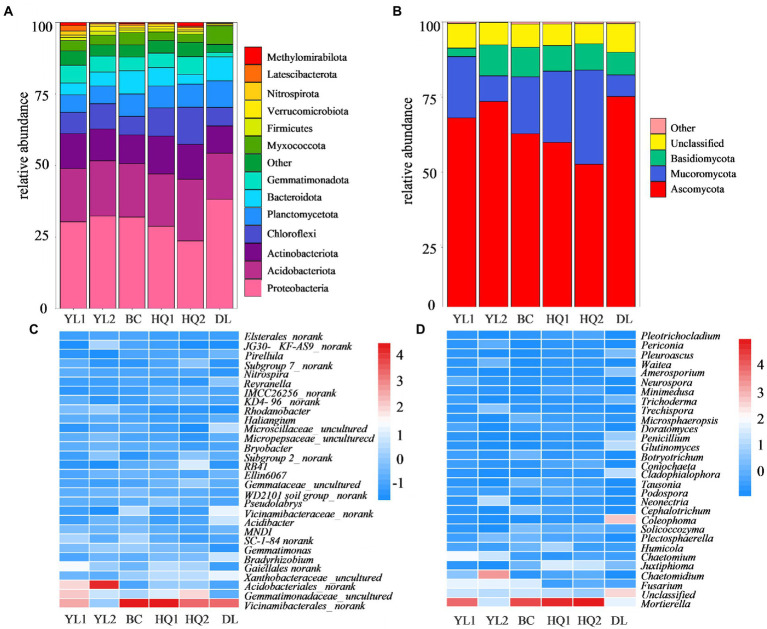
Relative abundances of the most abundant bacterial **(A)** and fungal **(B)** phyla and the top 30 bacterial **(C)** and fungal **(D)** genera.

A total of 105,756 bacterial and 16,734 fungal OTUs were obtained from the 1,078,669 and 1,107,370 quality-filtered sequences, respectively. For the bacteria, 13 phyla were detected with average relative abundances higher than 1%. Proteobacteria was the predominant phylum in most sample sites, with a relative abundance of 23.7–38.2%. The other most abundant bacterial phyla were Acidobacteriota (16.1–21.5%), Actinobacteriota (9.5–13.3%), and Chloroflexi (6.4–12.9%; Figure 3A). However, the relative abundance of these soil bacterial phyla differed among six sample sites. For instance, the relative abundances of Proteobacteria were consistently higher in DL compared to other sample sites (*p* = 0.023). In contrast, Acidobacteriota (*p* = 0.504), Actinobacteriota (*p* = 0.356), and Chloroflexi (*p* = 0.010) were higher in HQ1 and HQ2 than in other sample sites. Besides, at the general level, 2,068 taxa were identified across the sampled soils. The top 30 most abundant genera are shown in Figure 3C, and the dominant genera of these sample sites are similar, including *Vicinamibacterales_norank*, *Gemmatimonadaceae*_*uncultured*, *Acidobacteriales*_*norank*, *Xanthobacteraceae_uncultured*, and *Gaiellales*_*norank*.

Three fungal phyla have an abundance threshold more significant than 1% compared to bacteria and were included in community analysis. The relative abundances of the fungal phyla are displayed in Figure 3B. The results revealed that the fungal communities were dominated by Ascomycota, which comprised 52.6–75.2% of all identified sequences, followed by Mucoromycota (7.2–31.3%) and Basidiomycota (0.03–10.3%). Nevertheless, the relative abundances of these dominant phyla differed among the six sample sites in rhizosphere soil. In brief, the phylum Ascomycota showed the highest relative abundance in DL soils (*p* = 0.054), whereas the Mucoromycota (*p* = 0.015) and Basidiomycota (*p* = 0.271) were higher in HQ2 and YL2 than in other sample sites, respectively. Besides, 657 taxa were identified at the general level, and the relative abundance of dominant genera, including *Mortierella, Fusarium*, and *Chaetomidium*, and the top 30 most abundant genera are shown in Figure 3D.

### Identification of microbial biomarkers

**Figure 4:**
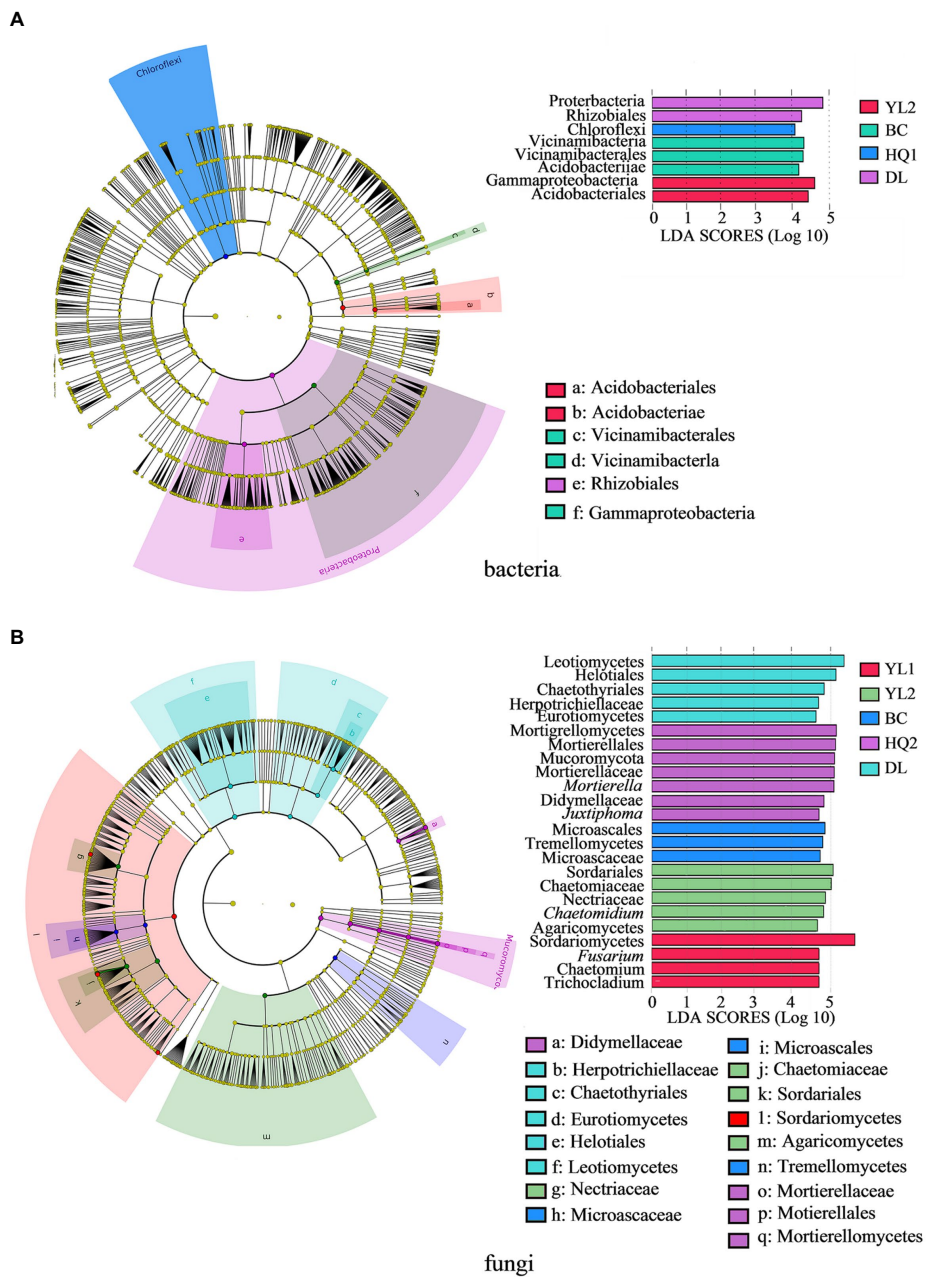
The LEfSe identifies the significantly different (*p* < 0.05, Kruskal-Wallis test, linear discriminant analysis values >4.0) bacteria **(A)** and fungi **(B)** at multiple taxonomic levels in the six regions. Colored dots represent the taxa with significantly different abundances between sites, and from the center outward, they represent the phylum, class, order, family, and genus levels.

The most differentially abundant taxonomic biomarker of bacteria and fungi was identified and compared using LEfSe analysis at the phylum and genus levels. Results indicated that four, seven, six, zero, eight, and seven biomarkers were identified in YL1, YL2, BC, HQ1, HQ2, and DL, respectively. For the bacterial community, eight biomarkers were discovered and classified into two phyla, three classes, and three orders (Figure 4A). At the phylum level, the relative abundance of Proteobacteria was notably enriched in DL, whereas Chloroflexi were more enriched in HQ2. At the class level, Acidobacteriae and Vicinamibacteria were enriched in BC, and Gammaproteobacteria was relatively abundant in YL2. At the order level, the dominant orders were Acidobacteriales and Vicinamibacterales in YL2 and BC, and the relative abundance of Rhizobiales was significantly enriched in DL.

Additionally, the findings of the LEfSe study indicated that 24 biomarkers were detected in the fungal community (Figure 4B). Six of these biomarkers were identified at the genus level. In contrast, the remaining taxa were identified as family (six), order (five), or higher levels (seven). Mucoromycota was the sole dominant biomarker at the phylum level, indicating that the sample sites did not significantly alter the phylum-level composition of fungal communities. At the class level, DL, BC, HQ2, YL2, and YL1 were found to have two, one, one, one, and one biomarker, respectively. It is noteworthy that HQ1 did not exclude any fungal biomarkers. Leotiomycetes and Eurotiomycetes were the most abundant biomarkers in DL, whereas Tremellomycetes, Mortierellomycete, Agaricomycetes, and Sordariomycetes were the respective dominant biomarkers in BC, HQ2, YL2, and YL1. Similar results were also found, showing that the DL was more enriched in fungal biomarkers than other locations at the order level. At the family level, only Herpotrichiellaceae was significantly more abundant in DL than in other sample locations. While Didymellaceae and Microascaceae were the main biomarkers in HQ2 and BC, respectively, the relative abundances of Chaetomiaceae and Nectriaceae were noticeably increased in the YL2. Moreover, six genera were significantly different in these sample sites, of which *Trichocladium*, *Chaetomium*, and *Fusarium* were enriched in YL1, *Mortierella*, *Juxtiphoma* were relatively abundant in HQ2, and *Chaetomium* served as a biomarker in BC. The microbial community structures significantly differed among the six sample sites at class and order levels.

### Soil physicochemical properties and bioactive ingredients analysis

The major physicochemical characteristics in the rhizosphere soil of six sample sites were summarized in Table 2. The Mantel test showed that except for NH^4+^, soil physicochemical properties, including pH, OM, AP, AK, B, Cu, Fe, Mn, and Zn, were significantly altered by sample sites (*p* < 0.05). In detail, the soil pH ranged from 4.96 to 6.02, indicating that this region is acidic soil. The content of AK and Cu in DL was lower than in other sites, whereas the Mn and NH^4+^ was the opposite. On average, the content of AK and Cu in DL was reduced by 20 and 1.5 times compared to the BC and HQ2 soil, respectively. In our study, the soil type at all sampling sites was yellow-brown-earth or black soil. The content of Fe was highest in black soil (HQ2) and lowest in yellow-brown-earth (HQ1), in the range of 135.14–224.20 mg·kg^−1^, indicating that even across the small sampling area, soil physicochemical properties were strongly influenced by soil type. As for soil nutrient properties, the content of OM ranked as HQ1 > HQ2 > DL > YL1 > YL2 > BC. Besides, the content of AP in BC was significantly higher than in other sites, while the content of B in HQ1 was significantly lower than in YL2. Notably, Zn was found in YL1, YL2, HQ2, and DL but was not detected in BC or HQ1.

**Table 2:** Soil physicochemical properties in different sample sites (means ± standard errors).

Sites	YL1	YL2	BC	HQ1	HQ2	DL
AK (mg·kg^−1^)	124.47 ± 0.00b	125.97 ± 30.87ab	288.54 ± 53.85a	177.1 ± 23.75ab	164.3 ± 48.50ab	11.85 ± 3.67b
NH^4+^ (mg·kg^−1^)	20.33 ± 9.32	49.81 ± 6.44	39.29 ± 26.53	42.01 ± 8.48	49.69 ± 3.46	54.28 ± 11.37
OM (mg·kg^−1^)	36.84 ± 1.35ab	36.41 ± 0.51ab	33.38 ± 3.22b	40.25 ± 1.47a	38.11 ± 1.16a	37.28 ± 0.62ab
pH	5.58 ± 0.19ab	4.96 ± 0.03b	6.02 ± 0.04ab	5.65 ± 0.16ab	5.81 ± 0.07ab	5.36 ± 0.32ab
AP (mg·kg^−1^)	56.64 ± 14.36bc	82.73 ± 2.63ab	106.57 ± 7.31a	39.37 ± 6.417bc	27.23 ± 2.80c	68.6 ± 26.86abc
Zn (mg·kg^−1^)	0.35 ± 0.37a	0.58 ± 0.30a	0.00 ± 0.00b	0.00 ± 0.00ab	0.21 ± 0.14a	0.41 ± 0.16a
Mn (mg·kg^−1^)	1.39 ± 0.06bc	0.70 ± 0.04c	2.68 ± 0.47ab	0.73 ± 0.06c	3.04 ± 0.30ab	4.51 ± 1.07a
Cu (mg·kg^−1^)	0.12 ± 0.01b	0.12 ± 0.00b	0.12 ± 0.00ab	0.15 ± 0.01a	0.14 ± 0.01ab	0.1 ± 0.01b
B (mg·kg^−1^)	3.73 ± 1.22ab	4.77 ± 0.59a	4.59 ± 0.28ab	2.26 ± 0.04b	2.42 ± 0.31ab	2.42 ± 0.09ab
Fe (mg·kg^−1^)	151.22 ± 3.57c	181.04 ± 8.16ab	162.55 ± 3.37b	135.14 ± 8.64d	224.20 ± 20.10a	190.13 ± 29.16ab

According to the Kruskal-Wallis test, different letters indicate significant differences (*p* < 0.05).

**Figure 5:**
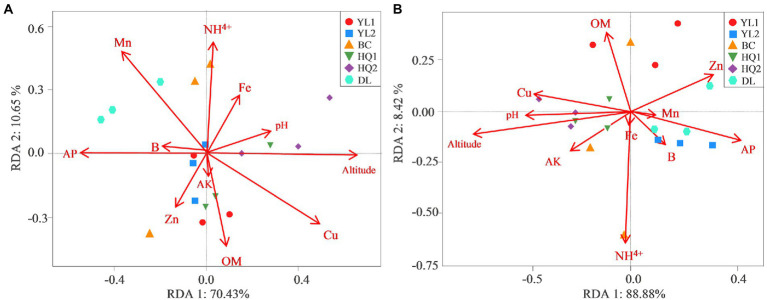
RDA measures the linkage between bacterial **(A)** and fungal **(B)** community composition, soil physicochemical properties, and altitude.

RDA was performed to test and visualize associations between soil properties and microbial community composition (Figure 5). The result showed that the first two axes explained more than 80% of bacterial and fungal community structure; RDA1 explained most of the variation. And altitude was the main factor affecting variance in bacterial and fungal communities as judged by the length of the vector, which has a significant positive relationship with microbial communities in HQ1 and HQ2, and a negative relationship with microbial communities in other sites. As the second important environmental variable, Cu was also a key factor affecting bacterial communities but showed less impact on fungal communities. For instance, Cu was positively correlated with bacterial communities in DL but was negatively correlated with HQ1 and HQ2. In contrast, NH^4+^, as another significant variable, primarily influenced the fungal community.

Besides, the Kruskal-Wallis test revealed that only benzoylaconine and benzoylhypaconine contents significantly differed among sample sites (Table 3). In brief, benzoylaconine was significantly different between YL1 and YL2 (*p* = 0.028) but was not significantly different between YL1 and other sites, implying that soil type had a more significant impact on benzoylaconine than sample sites. Moreover, the content of benzoylhypaconine was highest in YL2 (0.026 mg·g^−1^), and lowest in YL2 (0.0 mg·g^−1^), and the content of diester alkaloids and total alkaloids decreased in the following order: YL1 > YL2 > DL > BC > HQ1 > HQ2.

**Table 3:** Bioactive ingredients in different sample sites (means ± standard errors).

Bioactive ingredients	YL1	YL2	BC	HQ1	HQ2	DL
Benzoylmesaconine (mg·g^−1^)	0.0024 ± 0.002	0.0334 ± 0.020	0.0797 ± 0.083	0.043 ± 0.0138	0.044 ± 0.0302	0.04 ± 0.0163
Benzoylaconine (mg·g^−1^)	0.012 ± 0.001a	0.00 ± 0.00b	0.0085 ± 0.002ab	0.0061 ± 0.004ab	0.008 ± 0.002ab	0.010 ± 0.00ab
Benzoylhypaconine (mg·g^−1^)	0.026 ± 0.0062a	0.00 ± 0.00c	0.0092 ± 0.0074ab	0.0032 ± 0.0028abc	0.0011 ± 0.001bc	0.0048 ± 0.001abc
Monoester alkaloids (mg·g^−1^)	0.041 ± 0.008	0.033 ± 0.205	0.097 ± 0.093	0.052 ± 0.205	0.053 ± 0.033	0.064 ± 0.017
Mesaconitine (mg·g^−1^)	0.25 ± 0.033	0.7667 ± 0.576	0.6833 ± 0.185	0.37 ± 0.177	0.29 ± 0.100	0.65 ± 0.220
Aconitine (mg·g^−1^)	1.26 ± 0.297	0.11 ± 0.079	0.068 ± 0.058	0.04 ± 0.024	0.04 ± 0.008	0.091 ± 0.038
Hypaconitine (mg·g^−1^)	0.25 ± 0.034	0.11 ± 0.097	0.073 ± 0.058	0.043 ± 0.024	0.092 ± 0.008	0.11 ± 0.038
Diester alkaloids (mg·g^−1^)	1.76 ± 0.364	0.98 ± 0.750	0.82 ± 0.251	0.46 ± 0.211	0.43 ± 0075	0.85 ± 0.303
Total alkaloids (mg·g^−1^)	3.59 ± 0.742	2.03 ± 1.542	1.84 ± 0.486	1.01 ± 0.460	0.96 ± 0.211	1.82 ± 0.641

According to the Kruskal-Wallis test, different letters indicate significant differences (*p* < 0.05).

### Network analysis

**Figure 6:**
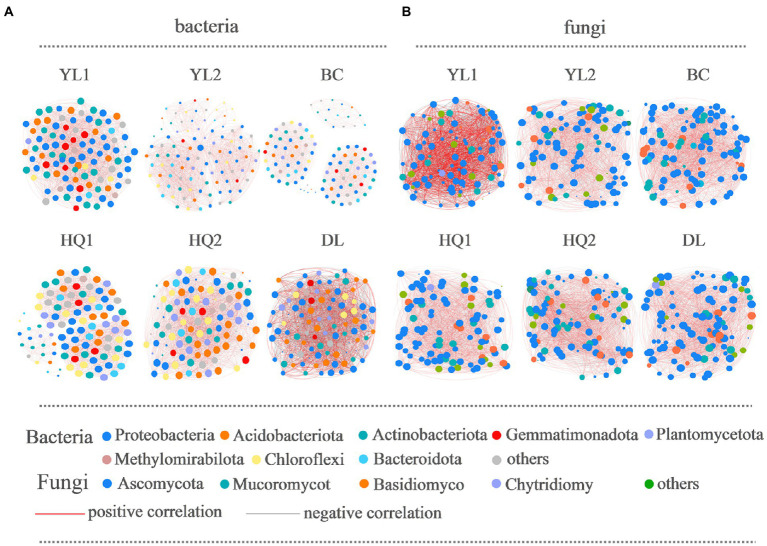
The co-occurrence network interactions of soil bacteria **(A)** and fungi **(B)**. Nodes represent OTUs, whereas a connection indicates a strong (Spearman’s |r| > 0.6) and significant positive (*p* < 0.05) correlation. The color of the nodes indicated the phylum, the size of each node was proportional to the number of connections (i.e., degree); the thickness of each connection between two nodes (i.e., edge) was proportional to the Spearman’s correlation coefficient (red indicates a positive correlation and gray indicates negative correlation).

Bacterial and fungal co-occurrence networks were constructed for the different sample sites (Figure 6), and topological properties were used to depict the complex patterns among microorganisms (Table 4). The results indicated that the bacterial and fungal networks significantly differed among these sample sites. Briefly, bacterial co-occurrence networks had fewer edges, lower density, and a lower average degree at YL1, HQ2, HQ1, BC, and DL than fungal networks, creating less complex rhizosphere network topologies. In detail, the nodes of OTUs in the bacterial network belonged mainly to Proteobacteria, Actinobacteria, and Acidobacteria, which was consistent with our results for community composition. In the HQ2 region, the number of edges, density, and average degree of the bacterial network significantly increased compared with the other rhizosphere soils. Co-occurrence networks had fewer negative correlations in YL1, YL2, HQ1, and HQ2 than in BC and DL, which can be interpreted as reduced inter-species competition.

**Table 4:** Network topological properties of bacterial and fungal communities in different sample sites.

	Taxonomy	YL1	YL2	BC	HQ1	HQ2	DL
Bacteria	Edges	1,569	1,550	1,631	1,418	1795	1,367
	Density	0.317	0.313	0.276	0.286	0.363	0.276
	Modularity	0.653	0.545	0.621	0.632	0.514	0.621
	Average degree	31.38	30.98	27.34	28.36	35.90	27.34
	Positive: Negative	1.20	1.28	0.98	0.96	1.05	1.04
Fungi	Edges	1,640	1,453	1769	1,519	1884	1,674
	Density	0.331	0.394	0.365	0.307	0.381	0.338
	Modularity	0.444	0.619	0.551	0.646	0.497	0.574
	Average degree	32.80	29.06	35.73	30.38	37.68	33.48
	Positive: Negative	1.01	1.02	1.34	1.08	1.01	1.01

For fungi, more than half of the nodes belonged to Ascomycota, Mucoromycot, and Basidiomycota; the rest belonged to Chytridiomy and others. These results were consistent with findings previously discussed and presented in Figure 3C. The relative abundance of Ascomycota was significantly different from the six sample sites, all ranked as DL > YL2 > HQ1 > BC > YL1 > HQ2, while Mucoromycot was the opposite, with 20, 14, 11, 11, 10, and 9% of nodes in HQ2, BC, YL1, HQ1, YL2, and DL, respectively. Besides, the numbers of edges, densities and average degrees of the fungal network in HQ2 were higher than those in other sites, while the modularity and density decreased. In contrast, the proportions of positive and negative connections in the YL1, YL2, HQ1, HQ2, and DL were similar but lower than BC.

### Distance decay of microbial community

**Figure 7:**
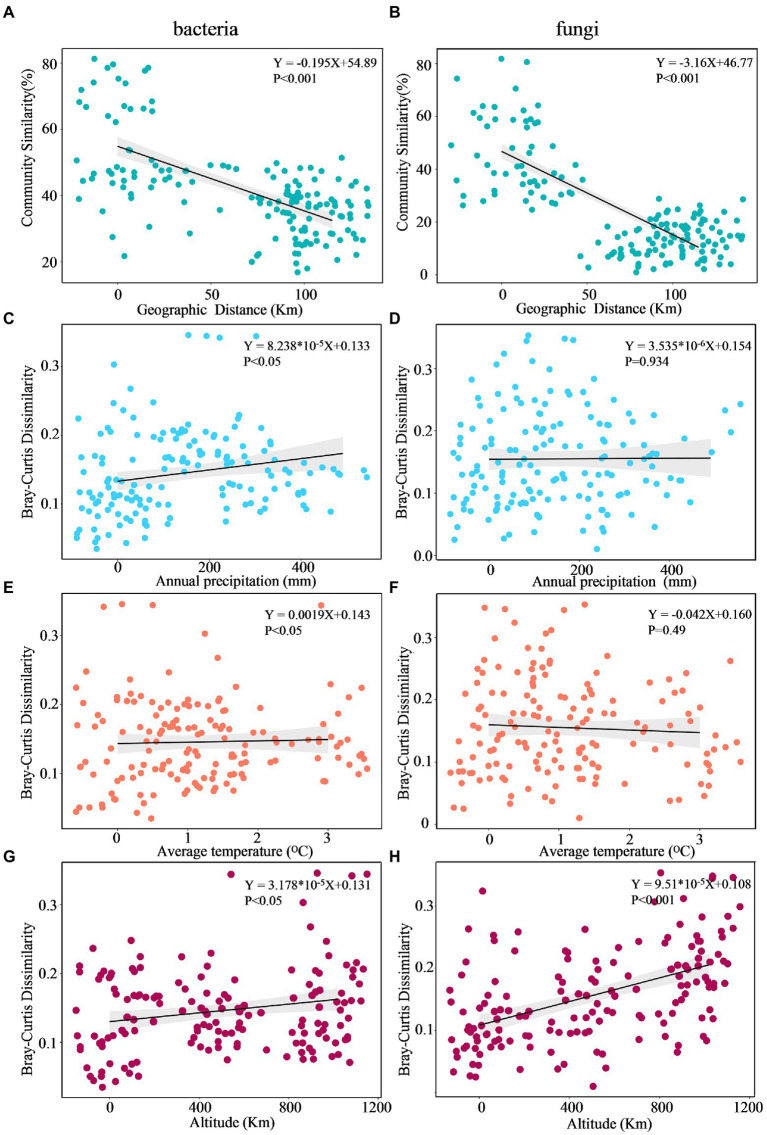
Multiple regression analysis of microbial community DDR across Euclidean distance **(A,B)**, annual precipitation **(C,D)**, average temperature **(E,F)**, and altitude **(G,H)**.

The DDR characterizes how community similarity changes across increasing geographical distances, and the results suggested that bacterial and fungal communities exhibited strong distance decay of similarity across the entire sampling range (Figure 7). Briefly, as geographic distance increased, bacterial and fungal communities became increasingly dissimilar for all samples. Specifically, fungi (*k* = −3.16) had a significantly higher turnover than bacteria (*k* = −0.195), with a steeper distance-decay relationship slope (Figures 7A,B). Besides, the slopes of geographical DDRs were significantly different from zero, and were steeper than those of environmental DDRs. Based on Mantel tests, the microbial community showed significant correlations with geographic distance (*r* = 0.238, *p* = 0.0128; *r* = 0.182, *p* = 0.036), it was not correlated with environmental variables (*r* = 0.0824, *p* = 0.219; *r* = 0.1887, *p* = 0.133). This finding suggests that at a geographic scale of 2–102 kilometers (km), geographic distance is more important than environmental variables in shaping the microbial community (Table 5).

**Table 5:** Spearman rank correlations (*r* values) of microbial community composition with matrices of environmental variables and geographic distance based on Mantel tests.

Organism	Parameter	Environmental variables	Geographic distance
Bacterial	Statistic *r*	0.0824	0.238
	Significance	0.219	0.0128^*^
Fungal	Statistic *r*	0.1887	0.182
	Significance	0.133	0.036^*^

* *p* < 0.05.

## Discussion

### Relationship between bacterial communities and environmental factors

Soil microorganisms play a crucial role in biogeochemical cycling (Zhou et al., 2014) and support the turnover and supply of nutrients for crop growth (Li et al., 2019), and their diversity and community composition can indirectly reflect soil fertility and biotransformation efficiency (Trivedi et al., 2013; Manasa et al., 2020). In this study, the dominant bacterial phyla were Proteobacteria, Actinobacteria, and Acidobacteriota in soil samples, consistent with results obtained from different soil environments (Carmen et al., 2019), which suggested that these bacteria exhibited great versatility in adapting to different environments (Feng et al., 2018; Tu et al., 2018). Similarly, Proteobacteria, Actinobacteria, and Acidobacteria were the most differentially abundant taxonomic biomarkers in LEfSe. Interestingly, recent studies have reported that Proteobacteria and Actinobacteria maintained higher metabolic activity in soil polluted by heavy metals (Margesin et al., 2011; He et al., 2017). For instance, the relative abundance of Proteobacteria was increased in the highly contaminated soil (Hu et al., 2021) and positively correlated with heavy metals Cu, Pb, and Cd (Zhao et al., 2020), whereas Actinobacteria abundance followed the opposite trend. These results demonstrated that the Proteobacteria were the metal-tolerant microorganisms in environments with high heavy metal contamination (Jiang et al., 2019; Li Y. C. et al., 2020). However, our study observed a negative correlation between Proteobacteria and Cu, and a positive correlation between Actinobacteriota and Cu (Supplementary Figure S1; Supplementary Table S1). The inconsistent results may be due to the different environmental conditions, which can influence the metabolic activity of microbial communities; that is, the microbial community structure in the soil varies with plant species and heavy metal pollution degree (Singh et al., 2014; Jayanthi et al., 2016). Besides, NH^4+^ is the most available form of nitrogen (N) in soil and can be directly taken up by plants. We found that Actinobacteria were weakly correlated with NH^4+^, yet the result was not consistent with those of Naether et al. (2012) and Ramirez et al. (2012), who found that the application of N consistently decreased the relative abundance of Actinobacteria. Further exploration is warranted to gain a more detailed understanding of the nature of observed relationships.

### Relationship between fungal communities and environmental factors

Consistent with previous studies, we found that Ascomycota was the dominant phylum among the sample sites, followed by Mucoromycota and Basidiomycota (Maestre et al., 2015; Prober et al., 2015; Porras Alfaro et al., 2017). Unlike bacteria, these fungal phyla were less responsive to heavy metals (Cu and Zn) in our study. Among them, Ascomycota was primarily positively influenced by OM (Li J. et al., 2022); they are typical “decomposers” that contribute to improving the OM decomposition and nutrient cycling of plants (Clemmensen et al., 2013; Zhou et al., 2016). In contrast, our results showed that Ascomycota was weakly correlated with OM (*r* = −0.186, *p* = 0.46), which might be related to the planting years. *A. carmichaelii* is an annual plant, and the soil generally features low OM content; the condition is less conducive to the growth of Ascomycota. Moreover, Li C. C. (2020) found that the relative abundance of Ascomycota tended to decline with increasing soil N substrate addition and was negatively correlated with available nitrogen, AP, and AK. In another study, Rojas et al. (2016) found a reduction in the phylum Ascomycota in soils with great P availability, and it could be that under P deficiency, there is an endophyte-specific change in fungal diversity and abundance. However, in our study, the abundance of Ascomycota was positively correlated with AP (*R*^2^ = 0.457, *p* = 0.058) and negatively correlated with AK (*R*^2^ = −0.416, *p* = 0.087). The inconsistent results may be related to using different experimental plants, soil types, and indigenous microorganisms. Of course, further study is needed to gain deep insight into potential correlations between Ascomycota, AP, and AK. Besides, AK was negatively correlated with the content of diester alkaloids and total alkaloids (Supplementary Figure S2; Supplementary Table S1), which was consistent with the previous study (Yue et al., 2014). In an experiment, Li Q. J. (2022) demonstrated that altitude had a marked effect on the abundances of Ascomycota, Basidiomycota, and Mucoromycota and that with increasing altitude, the relative abundance of Ascomycota showed a gradually decreasing trend. Our results were broadly consistent with those studies that altitude was positively correlated with Mucoromycota but negatively correlated with Ascomycota. However, not enough is yet understood about the ecological response and functional traits of these phyla.

### Co-occurrence network analysis of bacteria and fungi

Co-occurrence networks were influenced by sample sites, which provide insight into ecological interactions among microbial taxa, reveal ecological niches, and indicate keystone species (Berry and Widder, 2014; Zhao et al., 2019). In our study, the bacterial co-occurrence network showed fewer negative links in HQ1, which can be interpreted as reduced inter-species competition (Zelezniak et al., 2015; Fan et al., 2018); this phenomenon may be related to the carbon resource. These additional resources reduce competition in microbial communities, thus allowing more species to maintain free-living populations (Hubbell, 2005; Costello et al., 2012). Moreover, previous studies have shown that negative links may promote network stability as competition could buffer co-oscillation in microbial communities (Coyte et al., 2015; Zhou et al., 2020). Based on the above, we are more likely to believe that the structure of the bacterial network in HQ1 was more stable than in other soil sites, whereas the structure of the fungal network in BC was the opposite.

Modularity is another useful index to investigate the resistance of communities to disturbance (Zhou et al., 2011), which are clusters of densely interconnected nodes (Siles et al., 2021), and has been used to successfully predict the stability of networks of microbiomes (Herren and McMahon, 2017; De Vries et al., 2018). Krause et al. (2003) found that higher modularity was beneficial for increasing the stability of interaction networks and helping microbial communities resist environmental changes. In our study, the values of modularity in bacteria and fungi co-occurrence networks were greater than 0.4 in all sample sites, suggesting that distinct modules were formed in six sites (Newman, 2006; Li et al., 2021). Further, the HQ1 bacteria network showed higher modularity values than YL2, BC, HQ2, and DL, implying the increased stability of the HQ1 network.

The previous study proved that keystone species were critical in maintaining network stability and supporting soil functions (Lu et al., 2013; Fan et al., 2021). Our research confirmed that Proteobacteria, Acidobacteriota, Actinobacteriota, and Gemmatimonadota were keystone species in bacterial networks, whereas Ascomycota and Mucoromycot were keystone species in fungal networks. The relative abundances of these species were high, i.e., higher than 1%, which is inconsistent with previous findings (Li et al., 2021; Cheng et al., 2022), perhaps because of differences in root exudation. As shown in Figure 6, the proportion of nodes belonging to four bacterial phyla was ranked as YL1 > DL > BC > HQ1 > HQ2 > YL2, while both fungal phyla ranked in the order of BC > HQ2 > DL > YL2 > HQ1 > YL1. It’s generally known that more key taxa presented mean higher network stability (Coyte et al., 2015). According to the above discussion, we are more likely to believe that the network structure in the HQ1 soil was less stable than those of YL1, DL, and BC, contrary to the decreased negative links. The same trend was shown in the fungal network, which was more stable in the BC soil. These results suggested that a single parameter is not enough to predict the stability of the network, and a series of long-term experiments should be conducted to test it (Yuan et al., 2021).

In addition, only a few bacterial taxa, such as Proteobacteria, Actinobacteria, and Chloroflexi, have been linked to soil health (Schlatter et al., 2022). Among them, Proteobacteria prefer eutrophic and facultative anaerobic environments (Zhang et al., 2016; Yan et al., 2020), and are involved in disease suppression (Deng et al., 2020), whereas, Acidobacteria was favored in oligotrophic conditions with lower carbon availability and often used as an indicator of nutritional status (Naether et al., 2012; Jiang et al., 2017). Simultaneously, the ratio of Proteobacteria to Acidobacteria is an index of soil nutrients, positively related to increasing soil nutrients (Thomson et al., 2010). In the fungi community, Ascomycota is involved in the nutrient cycle to exploit nutrient-rich environments and can be associated with spreading soil disease (Guo et al., 2019). All this leads us to consider that further research on the specific taxa, microbial interactions, and the keystone taxa should provide a better picture of the function of the microbial network.

## Conclusion

Our findings highlight the differences in diversity, composition, keystone species, and DDR pattern of bacterial and fungal communities in rhizosphere soil among six sample regions. This study provides new insight for further understanding rhizosphere ecological function at a fine scale. Our results indicate that the Proteobacteria, Acidobacteriota, Ascomycota, and Mucoromycot OTUs were identified as highly abundant groups in all sample soils and categorized as keystone taxa in biological networks, where they played a critical role in soil health, N cycling, and disease suppression. Although the networks have been interpreted to predict microbial interactions, they remain a conceptual framework that needs further studies to verify the many predictions. From our experimental results, altitude was crucial in determining the composition of fungal and bacterial communities. Besides, bacterial and fungal communities exhibited strong distance decay of similarity at a geographic scale of 2–102 km, and geographic distance is more important than environmental variables in shaping the microbial community. Overall, this study enhances our understanding of the rhizosphere microbial communities and provides a theoretical basis and potential production strategies for planting *A. carmichaelii*.

## Data availability statement

The datasets presented in this study can be found in online repositories. The names of the repository/repositories and accession number(s) can be found at: https://www.ncbi.nlm.nih.gov/genbank/, PRJNA832898.

## Author contributions

All authors contributed to the study’s conception and design. BD, GW, JQ, CX, and TP conceived and designed the experiments. TP, JL, and JD performed the experiments. TP, JQ, and ZZ performed the data analysis. BD, GW, and TP wrote the manuscript. All authors contributed to the article and approved the submitted version.

## Funding

This work was supported by the key technology projects in Yunnan province of China (No. 201800501 and 202002AA100007), the Top talents of the Ten Thousand Talents Plan in Yunnan Province (YNWR-QNBJ-2020251), the Yunnan academician expert workstation (202205AF150026 and 202105AF150053), and the key technology projects in DL Bai Autonomous Prefecture (No. D2019NA03).

## Conflict of interest

The authors declare that the research was conducted in the absence of any commercial or financial relationships that could be construed as a potential conflict of interest.

## Publisher’s note

All claims expressed in this article are solely those of the authors and do not necessarily represent those of their affiliated organizations, or those of the publisher, the editors and the reviewers. Any product that may be evaluated in this article, or claim that may be made by its manufacturer, is not guaranteed or endorsed by the publisher.
