# Supplementary material for: Microbial community diversity and function analysis of Aconitum carmichaelii Debeaux in rhizosphere soil of farmlands in Southwest China
Source: Front Microbiol. 2022 Dec 15;13:1055638. doi: 10.3389/fmicb.2022.1055638 (PMC9797738; doi:10.3389/fmicb.2022.1055638)
Supplement: Supplementary file 1 [file Image_1.pdf]

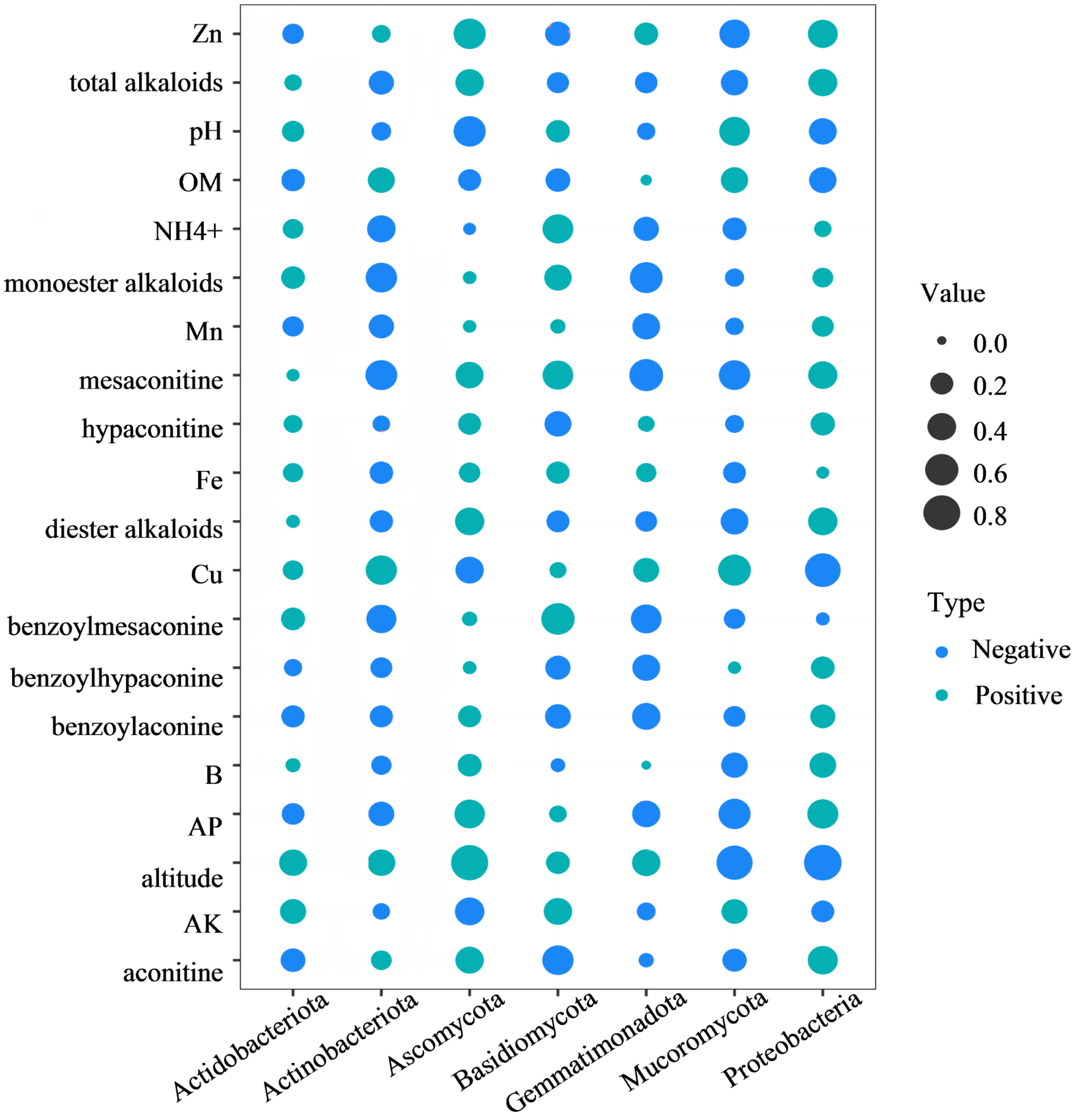


Fig. S1. Spearman correlation between the dominant bacterial and fungal phyla, bioactive ingredients, and soil physicochemical properties.
