# Supplementary material for: Microbial community diversity and function analysis of Aconitum carmichaelii Debeaux in rhizosphere soil of farmlands in Southwest China
Source: Front Microbiol. 2022 Dec 15;13:1055638. doi: 10.3389/fmicb.2022.1055638 (PMC9797738; doi:10.3389/fmicb.2022.1055638)
Supplement: Supplementary file 2 [file Image_2.pdf]

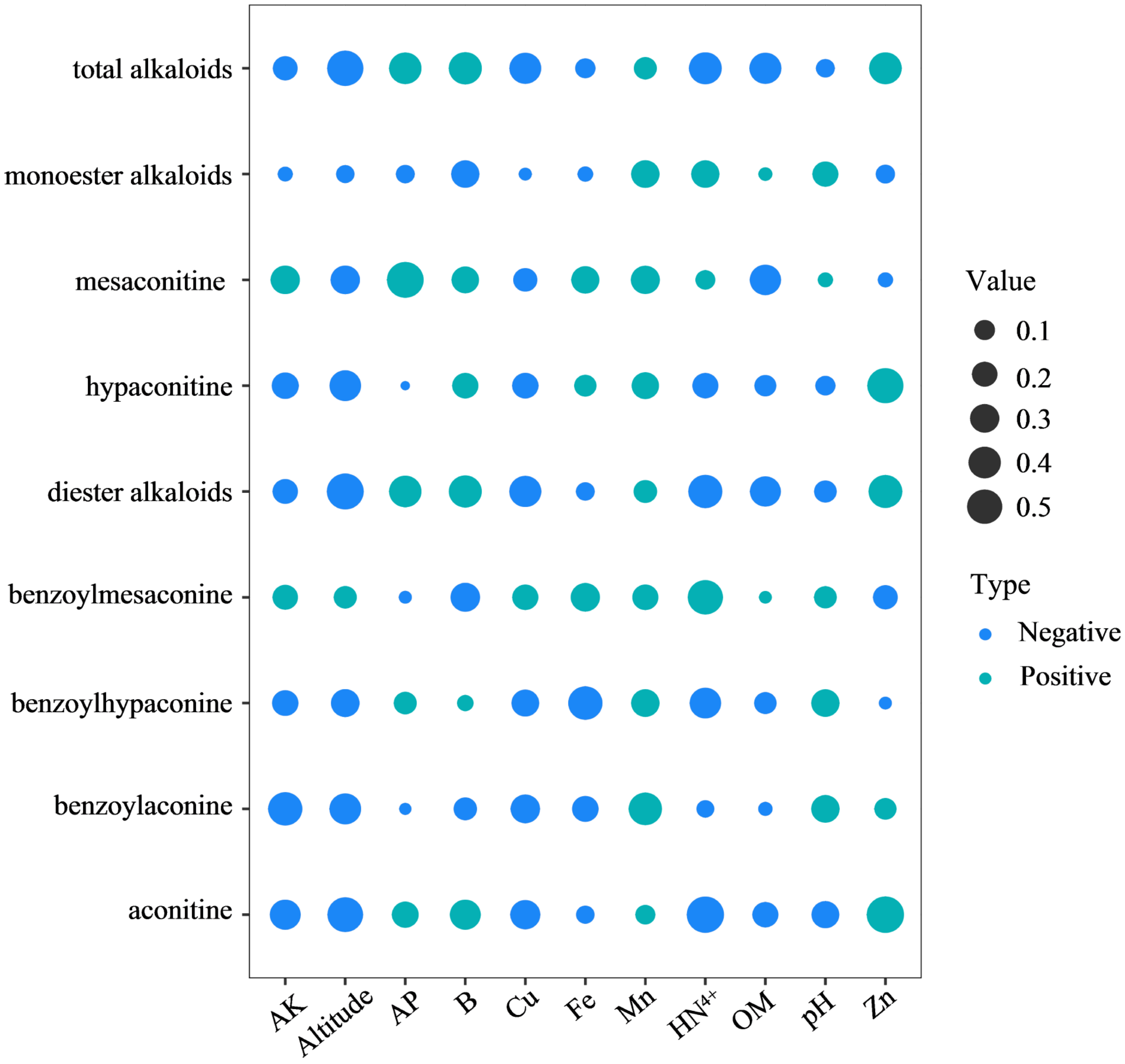

Fig. S2. Spearman correlation between the bioactive ingredients and soil physicochemical properties.
