# Supplementary material for: Microbial community diversity and function analysis of Aconitum carmichaelii Debeaux in rhizosphere soil of farmlands in Southwest China
Source: Front Microbiol. 2022 Dec 15;13:1055638. doi: 10.3389/fmicb.2022.1055638 (PMC9797738; doi:10.3389/fmicb.2022.1055638)
Supplement: Supplementary file 3 [file Table_1.docx]

Table S1. Spearman rank correlation analysis of dominant phylum, soil physicochemical properties, and bioactive ingredients. *P < 0.05, **P < 0.01.

| Parameter | Acidobacteriota | Actinobacteriota | Gemmatimonadota | Proteobacteria | Ascomycota | Basidiomycota | Mucoromycota |
| --- | --- | --- | --- | --- | --- | --- | --- |
| AK | 0.2921 | -0.0671 | -0.0918 | -0.1889 | -0.416 | 0.373 | 0.286 |
| NH^4+^ | 0.1331 | -0.3746 | -0.2632 | 0.0650 | -0.011 | 0.474* | -0.216 |
| OM | -0.2059 | 0.3166 | 0.0041 | -0.3301 | -0.186 | -0.240 | 0.331 |
| PH | 0.1683 | -0.1053 | -0.0795 | -0.3490* | -0.530 | 0.213 | 0.468 |
| AP | -0.1868 | -0.2817 | -0.3622 | 0.4861* | 0.457 | 0.071 | -0.534* |
| Zn | -0.1493 | 0.0893 | 0.2218 | 0.4341 | 0.536* | -0.275 | -0.453 |
| Mn | -0.1476 | -0.2570 | -0.3437 | 0.1641 | 0.015 | 0.036 | -0.082 |
| Cu | 0.1300 | 0.4843* | 0.2810 | -0.7139* | -0.369 | 0.058 | 0.566* |
| B | 0.0331 | -0.1229 | 0.0000 | 0.3120 | 0.217 | -0.028 | -0.308 |
| Fe | 0.1207 | -0.2074 | 0.1207 | 0.0114 | 0.146 | 0.201 | -0.183 |
| altitude | 0.3587 | 0.3587 | 0.3715 | -0.8258** | -0.741* | 0.215 | 0.798** |
| benzoylmesaconine | 0.2219 | -0.4365 | -0.4654 | -0.0196 | 0.036 | 0.595* | -0.148 |
| benzoylaconine | -0.2034 | -0.1972 | -0.3736 | 0.2553 | 0.193 | -0.280 | -0.163 |
| benzoylhypaconine | -0.0799 | -0.1609 | -0.3498 | 0.2138 | 0.019 | -0.259 | 0.015 |
| monoester alkaloids | 0.2157 | -0.4943* | -0.5542* | 0.1352 | 0.018 | 0.337 | -0.096 |
| mesaconitine | 0.0134 | -0.5108* | -0.6140* | 0.4056 | 0.356 | 0.461 | -0.498* |
| aconitine | -0.2487 | 0.1311 | -0.0341 | 0.4427 | 0.377 | -0.498* | -0.230 |
| hypaconitine | 0.0898 | -0.0691 | 0.0568 | 0.2322 | 0.189 | -0.317 | -0.090 |
| diester alkaloids | 0.0196 | -0.2033 | -0.1579 | 0.4138 | 0.406 | -0.191 | -0.344 |
| total alkaloids | 0.0671 | -0.2590 | -0.1765 | 0.3973 | 0.377 | -0.166 | -0.323 |
